# Comparative Efficacy of Selective Serotonin Reuptake Inhibitors (SSRIs) and Serotonin-Norepinephrine Reuptake Inhibitors (SNRIs) in the Management of Post-stroke Depression: A Systematic Review of Randomized Controlled Trials

**DOI:** 10.7759/cureus.84784

**Published:** 2025-05-25

**Authors:** Zarin Nudar Rodoshi, Sharen Shibu, Osman Omer, Hamza Tallal, Muhammad Zubair Dawud Gondal, Zayam Shahid, Ayesha Azam, Noor Abbas

**Affiliations:** 1 Medical Education, Mymensingh Medical College, Mymensingh, BGD; 2 Internal Medicine, Vinnytsia National Medical University, Vinnytsia, UKR; 3 Integrative Medicine, Prince Mohammed Bin Abdulaziz Hospital, Riyadh, SAU; 4 Internal Medicine, Robina Mubashar Hospital, Mirpur, PAK; 5 General Surgery, Robina Mubashar Hospital, Mirpur, PAK; 6 Internal Medicine, Railway General Hospital, Rawalpindi, PAK; 7 Internal Medicine, Services Hospital Lahore, Lahore, PAK

**Keywords:** antidepressants, duloxetine, escitalopram, post-stroke depression, randomized controlled trials, reboxetine, snris, ssris, systematic review, venlafaxine

## Abstract

Post-stroke depression (PSD) is a common neuropsychiatric complication that adversely affects rehabilitation outcomes, cognitive recovery, and quality of life in stroke survivors. While selective serotonin reuptake inhibitors (SSRIs) are widely used as first-line treatment, serotonin-norepinephrine reuptake inhibitors (SNRIs) have emerged as potential alternatives with broader neurochemical targets. This systematic review aimed to compare the efficacy of SSRIs and SNRIs in the treatment and prevention of PSD. A comprehensive literature search was conducted across PubMed, Embase, Scopus, and Google Scholar in accordance with PRISMA guidelines, applying filters for English-language clinical trials. Five randomized controlled trials met the inclusion criteria and were analyzed. The findings revealed that both SSRIs and SNRIs significantly improved depressive symptoms, with escitalopram showing early and superior antidepressant effects compared to sertraline. SNRIs like duloxetine and reboxetine demonstrated added benefits in cognitive outcomes, prevention of PSD, and symptom subtype-specific efficacy, particularly in retarded depression. While the overall risk of bias was low in most studies, limitations such as small sample sizes and limited direct head-to-head comparisons were noted. These results support the clinical utility of both drug classes and emphasize the need for individualized pharmacologic strategies based on patient characteristics and symptom profiles.

## Introduction and background

Post-stroke depression (PSD) is a prevalent neuropsychiatric complication affecting approximately one-third of stroke survivors, significantly hindering rehabilitation outcomes and overall quality of life [1]. The onset of depression after stroke not only compromises functional recovery but also increases morbidity, mortality, and the likelihood of recurrent cerebrovascular events [2]. The pathophysiology of PSD is multifactorial, involving a complex interplay of neurochemical imbalances, structural brain damage, inflammation, and psychosocial stressors. Among the neurobiological contributors, alterations in serotonergic and noradrenergic pathways play a central role, highlighting the therapeutic importance of targeting these systems in pharmacological management [3,4].

Selective serotonin reuptake inhibitors (SSRIs) are frequently recommended as first-line agents in treating PSD due to their favorable side effect profile and established efficacy in general depressive disorders [5]. SSRIs function by selectively inhibiting the reuptake of serotonin (5-HT) into presynaptic neurons, thereby increasing its availability in the synaptic cleft and enhancing serotonergic neurotransmission. Commonly used SSRIs such as fluoxetine, escitalopram, sertraline, and citalopram have been widely studied in both psychiatric and neurological populations, with some trials focusing specifically on their role in PSD [6].

On the other hand, serotonin-norepinephrine reuptake inhibitors (SNRIs) like venlafaxine and duloxetine have emerged as promising alternatives, particularly for patients who demonstrate inadequate response or tolerability to SSRIs. SNRIs inhibit the reuptake of both serotonin and norepinephrine, potentially offering a broader therapeutic effect by influencing additional neurotransmitter pathways involved in mood regulation, attention, and cognitive function. By modulating both serotonergic and noradrenergic systems, SNRIs may offer theoretical advantages in addressing the broader spectrum of affective and cognitive symptoms associated with PSD [7].

Despite the growing interest in both drug classes, there remains a lack of consensus on whether SSRIs or SNRIs provide superior outcomes in the management of PSD. Many studies have focused on the individual efficacy of either SSRIs or SNRIs, yet direct comparative evidence remains limited and scattered across trials with varying methodologies, populations, and outcome measures. Consequently, a comprehensive synthesis of the available evidence is necessary to inform clinical decision-making and optimize pharmacological strategies for post-stroke patients experiencing depressive symptoms.

This systematic review aims to address this gap by evaluating and comparing the efficacy of SSRIs versus SNRIs in the treatment of post-stroke depression. Using the PICO framework [8], the population (P) includes adult patients diagnosed with PSD, the intervention (I) involves treatment with SSRIs, the comparator (C) consists of SNRIs, and the outcomes (O) assessed include depression symptom improvement, functional recovery, and incidence of adverse effects.

## Review

Materials and methods

Search Strategy

A comprehensive literature search was conducted in accordance with PRISMA (Preferred Reporting Items for Systematic Reviews and Meta-Analyses) guidelines [9] to identify relevant studies comparing the efficacy of SSRIs and SNRIs in PSD. Databases searched included PubMed, Scopus, Embase, and Google Scholar, covering articles published up to 2024. The search strategy utilized a combination of MeSH terms and free-text keywords such as “post-stroke depression,” “SSRIs,” “SNRIs,” “antidepressants,” and specific drug names like “fluoxetine,” “venlafaxine,” “duloxetine,” “escitalopram,” and “reboxetine.” Filters were applied to limit results to English-language articles and clinical trials involving human subjects. Only randomized controlled trials or prospective studies evaluating pharmacological interventions in PSD were included. Duplicates were removed, and titles and abstracts were screened for relevance before full-text review and final inclusion based on the eligibility criteria.

Eligibility Criteria

Studies were deemed eligible for inclusion if they met predefined criteria based on the PICO framework. The population included adult patients diagnosed with PSD, regardless of stroke type or duration since onset. Eligible interventions were SSRIs, such as fluoxetine, escitalopram, sertraline, and citalopram, while comparators included SNRIs such as venlafaxine, duloxetine, and reboxetine. Only clinical trials that provided head-to-head comparisons between SSRIs and SNRIs or evaluated either class in the context of PSD were considered. Studies had to be published in English and classified as clinical trials. Case reports, reviews, animal studies, and studies without measurable outcomes related to depression severity or functional recovery were excluded. Although observational studies may offer additional clinical perspectives, they were excluded to maintain methodological rigor, reduce bias, and focus the analysis on high-quality evidence from randomized controlled trials.

Data Extraction

Data extraction was performed independently by two reviewers using a standardized template to ensure consistency and accuracy. Key information extracted from each study included authorship, year of publication, study design, sample size and population characteristics, intervention and comparator details (including drug names and dosages), treatment duration, outcome measures used (e.g., HAMD, BDI, TAS-20, MMSE, ADL), and major findings. Where available, statistical results such as p-values, mean score changes, and confidence intervals were recorded to facilitate quantitative and qualitative comparison. Any discrepancies during extraction were resolved by discussion and consensus among the reviewers.

Data Analysis and Synthesis

Given the heterogeneity in study design, outcome measures, and comparator drugs, a narrative synthesis approach was employed. Studies were grouped based on drug class comparisons and analyzed for patterns in efficacy, response onset, functional outcomes, and subgroup-specific effects. Emphasis was placed on both direct comparisons (e.g., SSRI vs. SNRI) and clinically significant trends such as differential responses in anxious versus retarded depression. Statistical outcomes from each study were interpreted within context, and the overall quality of evidence was assessed using the Cochrane Risk of Bias 2.0 tool [10] to guide the reliability of the synthesized findings.

Results

Study Selection Process

**Figure 1:**
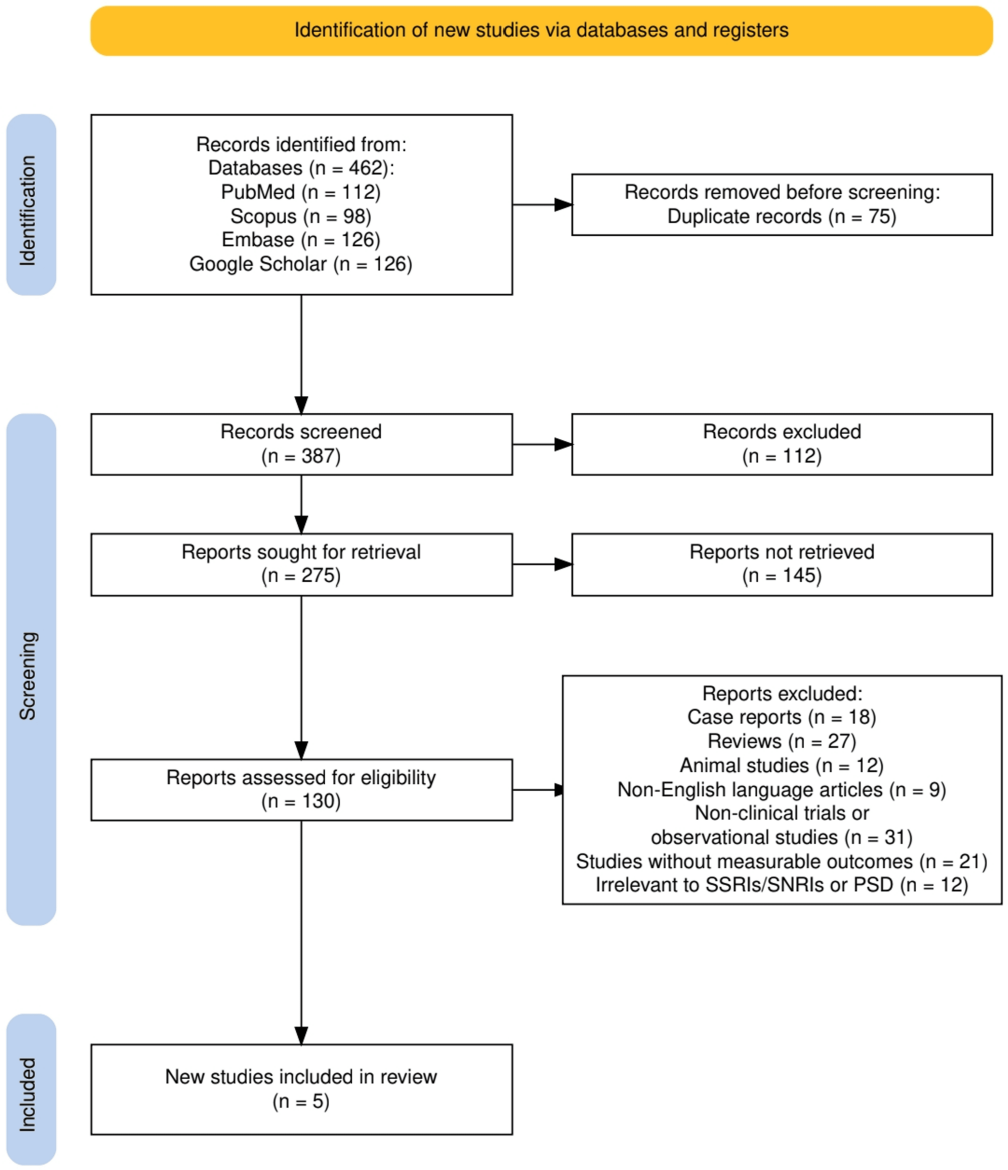
The PRISMA flowchart represents the study selection process. PRISMA: Preferred Reporting Items for Systematic reviews and Meta-Analyses; SSRIs: Selective Serotonin Reuptake Inhibitors; SNRIs: Serotonin-Norepinephrine Reuptake Inhibitors; PSD: Post-stroke Depression

The study selection process is summarized in Figure 1, which outlines the PRISMA flow diagram used to document each step of article identification and screening. A total of 462 records were retrieved through database searches: PubMed (112), Scopus (98), Embase (126), and Google Scholar (126). After removing 75 duplicate entries, 387 articles were screened based on titles and abstracts. From these, 112 records were excluded for not meeting the basic relevance criteria. Of the 275 full-text articles sought for retrieval, 145 could not be accessed, leaving 130 studies for full-text eligibility assessment. During this phase, 130 studies were evaluated, and 125 were excluded based on predefined eligibility criteria: case reports (18), reviews (27), animal studies (12), non-English articles (9), non-clinical trials or observational studies (31), studies lacking measurable outcomes (21), and those irrelevant to SSRIs, SNRIs, or PSD (12). Ultimately, five studies met the inclusion criteria and were included in the final systematic review.

Characteristics of the Selected Studies

As shown in Table 1, the five included studies comprised randomized controlled trials evaluating the comparative efficacy of SSRIs and SNRIs in patients with PSD. Sample sizes ranged from 50 to 95 participants, and patient populations included both general adult stroke survivors and elderly individuals with specific depressive subtypes. The interventions examined involved widely used SSRIs such as escitalopram, fluoxetine, and citalopram, while the comparators included SNRIs like duloxetine, venlafaxine, and reboxetine. Treatment durations spanned from 8 to 16 weeks, and a variety of standardized outcome measures were employed across studies, including HAMD, HDRS, BDI, MOCA, MMSE, ADL, and TAS-20. The findings consistently demonstrated the efficacy of both drug classes in alleviating depressive symptoms, with certain agents showing distinct advantages in specific symptom domains such as emotional unawareness or subtype-specific response patterns. Statistical significance was reported in most studies, strengthening the reliability of the observed effects.

**Table 1:** The summary of all the selected studies in the systematic review. RCT: Randomized Controlled Trial; ICD-10: International Classification of Diseases, 10th Revision; PSD: Post-stroke Depression; SSRI: Selective Serotonin Reuptake Inhibitor; SNRI: Serotonin-Norepinephrine Reuptake Inhibitor; HAMD-24: 24-Item Hamilton Depression Rating Scale; HAMA-14: 14-Item Hamilton Anxiety Rating Scale; MOCA: Montreal Cognitive Assessment; ADL: Activities of Daily Living; NIHSS: National Institutes of Health Stroke Scale; MMSE: Mini-Mental State Examination; SF-36: 36-Item Short Form Survey (Health-Related Quality of Life); DSM-IV: Diagnostic and Statistical Manual of Mental Disorders, 4th Edition; TAS-20: Toronto Alexithymia Scale (20-item); HDRS: Hamilton Depression Rating Scale; BDI: Beck Depression Inventory; BID: Bis in Die (Twice Daily)

Study (Author, Year)	Study Design	Sample Size & Population	Intervention (SSRI)	Comparator (SSRI/SNRI)	Duration of Treatment	Outcome Measures	Key Findings	Statistical Data
Yan & Hu, 2024 [11]	RCT	n = 60; Adults (40–89 yrs) with ICD-10 diagnosed PSD	Escitalopram (10–20 mg/day, n = 30)	Sertraline (50–200 mg/day, n = 30)	8 weeks	HAMD-24, HAMA-14, MOCA, ADL	Escitalopram superior in reducing depression; both drugs comparable in anxiety, cognition, and ADL improvement	HAMD-24 post-treatment: p < 0.05; Week 1 difference: p < 0.01; HAMA-14: p > 0.05
Zhang et al., 2013 [12]	Open single-blind RCT	n = 95; Ischemic stroke patients without baseline depression	—	Duloxetine (30–90 mg/day, n = 47) vs. routine therapy (n = 48)	12 weeks treatment; 24 weeks follow-up	Hamilton Depression Scale, NIHSS, MMSE, ADL, SF-36	Duloxetine reduced incidence of both minor and major PSD by 16%; improved cognitive function, QoL, and stroke recovery	PSD incidence ↓16% in duloxetine group; MMSE and SF-36 improvements noted (exact p-values not provided)
Cravello et al., 2009 [13]	RCT (open-label)	n = 50; Inpatients with first-ever stroke and DSM-IV PSD	Fluoxetine (20–40 mg/day, n = 25)	Venlafaxine SR (75–150 mg/day, n = 25)	8 weeks	HAMD, MMSE, TAS-20	Both groups improved in depression; venlafaxine significantly better at reducing alexithymia severity	Greater TAS-20 reduction in venlafaxine group (p < 0.05); similar HAMD improvements between groups
Rampello et al., 2004 [14]	RCT (double-blind)	n = 74; PSD patients with anxious or retarded depression	Citalopram (50% of each subgroup)	Reboxetine (50% of each subgroup)	16 weeks	HDRS, BDI, Synoptic table	Citalopram better in anxious PSD; reboxetine better in retarded PSD; both well-tolerated	Subgroup analysis: citalopram > reboxetine in anxious PSD (p < 0.05); reverse true in retarded PSD
Rampello et al., 2005 [15]	RCT (double-blind, placebo-controlled)	n = elderly PSD patients with retarded depression	—	Reboxetine 4 mg BID vs. placebo	16 weeks	HDRS, BDI	Reboxetine significantly improved depression vs. placebo; well tolerated in elderly with PSD	HDRS ↓ from 24.06±1.52 to 9.26±2.15; BDI ↓ from 20.56±2.16 to 8.06±3.43; p < 0.01 vs baseline and placebo

Quality Assessment

As outlined in Table 2, the risk of bias assessment using the Cochrane RoB 2.0 tool indicates that the overall quality of the included studies is moderate to high. Three of the five studies, Yan & Hu [11], Rampello et al. [14], and Rampello et al. [15], were assessed as having a low risk of bias across most domains, including randomization process, deviations from intended interventions, missing outcome data, and outcome measurement. These trials demonstrated methodological rigor and transparency in reporting. In contrast, Zhang et al. [12] and Cravello et al. [13] were rated as having “some concerns” due to issues related to study design, such as open-label formats, limited blinding, and unclear reporting on prespecified outcomes. Despite these concerns, the studies maintained low risk in critical areas such as missing data and outcome measurement, allowing their inclusion in the final synthesis while acknowledging potential limitations in interpretability.

**Table 2:** The quality assessment of all the included studies.

Study (Author, Year)	Randomization Process	Deviations from Intended Interventions	Missing Outcome Data	Measurement of Outcome	Selection of Reported Results	Overall Risk of Bias
Yan & Hu, 2024 [11]	Low Risk	Low Risk	Low Risk	Low Risk	Low Risk	Low Risk
Zhang et al., 2013 [12]	Some Concerns	Some Concerns	Low Risk	Some Concerns	Some Concerns	Some Concerns
Cravello et al., 2009 [13]	Some Concerns	Some Concerns	Low Risk	Low Risk	Some Concerns	Some Concerns
Rampello et al., 2004 [14]	Low Risk	Low Risk	Low Risk	Low Risk	Some Concerns	Low Risk
Rampello et al., 2005 [15]	Low Risk	Low Risk	Low Risk	Low Risk	Low Risk	Low Risk

Discussion

The findings of this systematic review suggest that both SSRIs and SNRIs demonstrate meaningful efficacy in managing PSD, with some agents showing specific advantages. In a randomized controlled trial by Yan and Hu [11], escitalopram significantly reduced depressive symptoms compared to sertraline, with HAMD-24 scores showing a greater reduction in the escitalopram group by the first week (p < 0.01), and a statistically significant difference at eight weeks (p < 0.05), although anxiety, cognition, and ADL outcomes were comparable between both SSRIs. Zhang et al. [12] reported that duloxetine, an SNRI, reduced the incidence of PSD by 16% and enhanced recovery and cognitive function over a 24-week follow-up, indicating potential prophylactic benefits, although exact p-values were not disclosed. Cravello et al. [13] compared venlafaxine (SNRI) to fluoxetine (SSRI) and found both effective in treating depression, but venlafaxine was significantly superior in improving alexithymia severity (p < 0.05). Subgroup analysis in the study by Rampello et al. [14] revealed that citalopram SSRI was more effective in anxious depression, whereas reboxetine (SNRI) was more beneficial in retarded depression (p < 0.05 for both comparisons). Additionally, Rampello et al. [15] demonstrated that reboxetine led to a marked reduction in depression scores among elderly PSD patients with retarded features, with HDRS scores decreasing from 24.06±1.52 to 9.26±2.15 and BDI scores from 20.56±2.16 to 8.06±3.43 (p < 0.01 vs. baseline and placebo). Overall, while SSRIs remain effective first-line agents, SNRIs may offer targeted advantages in specific clinical profiles, particularly in patients with emotional unawareness or retarded depressive features. The findings of this review largely align with existing literature on the pharmacologic management of PSD, reinforcing the efficacy of SSRIs as a first-line treatment while highlighting the potential benefits of SNRIs in certain contexts. Previous meta-analyses and guidelines have consistently supported the use of SSRIs such as fluoxetine, sertraline, and escitalopram for PSD [16], citing their safety and tolerability. The current evidence corroborates this, particularly with Yan & Hu’s [11] results showing escitalopram’s superior antidepressant effect over sertraline. However, this review also extends existing knowledge by shedding light on the distinct advantages of SNRIs, such as duloxetine’s role in PSD prevention and venlafaxine’s benefit in emotional processing (alexithymia), which have been underexplored in earlier studies. While SSRIs are more frequently studied and prescribed, the consistent positive outcomes observed with SNRIs across multiple trials suggest that they may warrant broader clinical consideration and further head-to-head comparisons. The differential responses observed between SSRIs and SNRIs in this review may be explained by their distinct pharmacodynamic profiles and the neurochemical basis of PSD. SSRIs primarily enhance serotonergic transmission, which is known to regulate mood and emotional well-being [17]. SNRIs, on the other hand, act on both serotonin and norepinephrine pathways, which are additionally involved in attention, arousal, and motivation, domains often impaired after stroke [18]. This dual mechanism may explain why SNRIs like venlafaxine showed superior outcomes in addressing emotional unawareness (alexithymia) in the study by Cravello et al. [13], and why duloxetine demonstrated preventive benefits in PSD development. Moreover, norepinephrine’s role in modulating executive function and response to stress could underlie the observed improvements in cognitive recovery and quality of life among SNRI-treated patients, suggesting a mechanistically plausible advantage over SSRIs in broader neurobehavioral rehabilitation [19,20]. Notably, this review identified clinically relevant differences in treatment response among PSD subtypes, underscoring the importance of individualized therapy. The study by Rampello et al. [14] is particularly illuminating, demonstrating that patients with anxious depression responded better to citalopram (an SSRI), while those with retarded depression exhibited more improvement with reboxetine (an SNRI). This distinction highlights how symptom profile and depression subtype can influence pharmacological outcomes. Similarly, Rampello et al. [15] showed that elderly patients with retarded depression benefited significantly from reboxetine compared to placebo, suggesting that age and clinical presentation are important modifiers of treatment efficacy. These findings advocate for a more stratified approach in PSD management, tailoring antidepressant selection based on patient characteristics rather than a one-size-fits-all model [21,22].

This review demonstrates methodological strength through its adherence to PRISMA guidelines, use of a well-defined PICO framework, and selection of high-quality randomized controlled trials with predominantly low risk of bias. The included studies provided direct and relevant comparisons between SSRIs and SNRIs in PSD, allowing for a focused synthesis of clinical outcomes. The narrative structure with clearly defined subheadings further supports the logical flow of information and facilitates accessibility for both clinical and academic audiences.

However, several limitations must be acknowledged. The primary constraint is the limited number of randomized controlled trials directly comparing SSRIs and SNRIs, which restricts the depth of comparative analysis. Additionally, variability in study design-such as open-label or single-blind formats-may have introduced performance or detection bias. Small sample sizes in individual trials and inconsistent reporting of subgroup analyses (e.g., depression subtype, stroke severity) further reduce the generalizability of findings. Given the scarcity of head-to-head RCTs, future reviews may consider incorporating alternative methodologies such as high-quality observational studies, real-world data registries, or network meta-analyses to provide broader comparative insight. Although these were not included in the current review to preserve methodological rigor, their inclusion in future research could enhance evidence synthesis and better inform personalized treatment strategies for PSD.

The findings of this review have important clinical and research implications for the management of PSD. Clinically, while SSRIs remain the preferred first-line agents due to their well-established safety and efficacy profiles, SNRIs may offer targeted advantages in specific patient populations [23]. For instance, venlafaxine appears more effective in patients with prominent alexithymia [24], and reboxetine shows greater benefit in those with retarded depression, particularly among the elderly. These insights support a more individualized, symptom-driven approach to antidepressant selection in PSD [25]. From a research perspective, the current literature reveals a lack of large-scale, multicenter trials directly comparing SSRIs and SNRIs with standardized outcome measures. There is also limited data on long-term efficacy, relapse prevention, and quality-of-life outcomes. Future studies should address these gaps, focusing on stratified analyses by depression subtype, age group, and stroke characteristics to refine treatment guidelines and improve personalized care in this vulnerable population.

## Conclusions

This systematic review offers a focused comparison of SSRIs and SNRIs in the treatment of PSD, highlighting the nuanced advantages of each pharmacologic class. While SSRIs continue to be foundational in PSD management, SNRIs demonstrate promising efficacy in specific clinical contexts, such as emotional unawareness and retarded depressive subtypes. By synthesizing current evidence from randomized controlled trials, this review reinforces the importance of individualized treatment strategies and contributes to a more refined understanding of antidepressant selection in post-stroke care. These findings not only support evidence-based decision-making but also underscore the need for continued research to optimize outcomes in this complex and often under-recognized condition.
